# Chemical Drivers of Sweetness, Sourness, and Astringency in Apple Juice Identified Using Support Vector Machine Regression

**DOI:** 10.1111/1750-3841.71479

**Published:** 2026-09-16

**Authors:** Annachiara Berardinelli, Danny Cliceri, Isabella Endrizzi, Flavia Gasperi, Urska Vrhovsek, Eugenio Aprea

**Affiliations:** ^1^ Department of Industrial Engineering University of Trento Povo Trento Italy; ^2^ Center Agriculture Food Environment University of Trento San Michele all Adige Trento Italy; ^3^ Edmund Mach Foundation Research and Innovation Centre San Michele all Adige Trento Italy

## Abstract

The role of the main apple compositional parameters (sugars, organic acids, polyphenols, and volatile organic compounds) and their interactions on the perceived sensory attributes of Sweetness, Sourness, and Astringency was investigated in apple juice. Bivariate correlations and Support Vector Machine Regression (SVMR) analyses were used to examine the relationships between all the analyzed features and their relative importance in an interactive mode. By analyzing the contribution of the features to the SVMR models using Shapley values, we found that sweetness was strongly influenced by sucrose and by a few volatile organic compounds (VOCs), including acetate and butyl esters associated with fruity odors and flavors. For sourness, as expected, a strong contribution is given by malic acid, while VOCs associated with fruity odor contribute negatively. Regarding astringency, the major contributions were given by malic acid, procyanidins, sorbitol, and chlorogenic acid; the VOCs (few esters, hexanol, and (E)‐2‐hexenal) show a suppressing effect on astringency. In our investigation, SVMR confirms the role and the importance of the evidenced compositional parameters in driving the perception of the three analyzed sensory attributes in apple juices.

**Practical Applications:**

This research could help provide apple breeders with new varieties with more predictable flavor, such as sweeter apples for juice or more tannic apples for cider. It could also help juice and cider producers select, blend, and process apples more consistently by using chemical measurements to estimate how a product may taste. The work may support better quality control, reduces variability, and strengthen product differentiation in markets where flavor consistency and clean‐label formulations are increasingly important.

## 1 Introduction

Consumer preference for apples and apple juices is primarily driven by sensory perception. Sweetness, fruitiness, and floral notes positively influence acceptability, whereas excessive sourness, astringency, and vegetal flavors reduce preference (Charles et al. 2018). In apple juices in particular, products characterized by fresh and green odors combined with moderate sweetness and reduced sourness and astringency are generally preferred (Okayasuand and Naito 2001). These observations highlight that apple quality is perceived through a complex sensory profile rather than through single attributes considered in isolation.

Although sweetness is commonly associated with sugar content, the relationship between chemical composition and perceived sweetness in apples is not straightforward. Apples contain several sugars and sugar alcohols, mainly sucrose, fructose, glucose, xylose, and sorbitol, and soluble solids content (SSC, °Brix) is often used as a practical indicator of sweetness (Oraguzie et al. 2009; Zhang et al. 2010). However, previous studies have shown only weak to moderate correlations between SSC and perceived sweetness, indicating that differences in taste perception become evident only beyond relatively large changes in °Brix and that sensory evaluation remains essential (Harker et al. 2002; Harker et al. 2006). These findings suggest that sweetness perception cannot be reliably predicted by sugar content alone.

Beyond sugars and acids, sensory perception in apple products is strongly influenced by multisensory interactions involving VOCs. Odor‐induced modulation of taste perception is a well‐established phenomenon mediated by central nervous system integration and associative learning (Spence 2022; Stevenson et al. 1998). Although apples contain hundreds of VOCs, only a limited subset, mainly esters and other aroma‐active molecules, has been shown to significantly enhance perceived sweetness through cross‐modal interactions (Barba et al. 2018; Goff and Klee 2006; Knoop et al. 2008). Previous multivariate studies showed that combinations of VOCs, particularly those associated with fruity odors, explain part of sweetness perception together with sugar or acid content, whereas odors not associated with sweetness reduce its perception (Aprea et al. 2017). Together, these results reinforce the idea that sensory attributes emerge from interactions among multiple chemical drivers.

While multisensory interactions have been extensively investigated for sweetness, comparable integrative approaches remain limited for other key sensory attributes such as Sourness and Astringency, particularly in complex food matrices such as fruit juices. Sourness perception is primarily related to organic acid composition and concentration, but it is also modulated by sugars and aroma compounds through mixture suppression and cross‐modal effects, complicating its prediction (Keast and Breslin 2003; Shankar et al. 2010). Recent studies have further demonstrated that aroma–taste interactions can significantly alter sourness perception, highlighting the need for multivariate approaches that jointly consider chemical and sensory dimensions (Cliceri et al. 2021). Astringency is mainly associated with phenolic compounds, particularly flavanols and other tannin‐related structures, yet its sensory mechanisms and interactions with aroma and taste remain only partially understood (Gibbins and Carpenter 2013; Shanker et al. 2024). Although emerging evidence indicates that VOCs may modulate astringency intensity and persistence, these effects have been explored predominantly in simplified model systems rather than in real food matrices (Ferrer‐Gallego et al. 2014; Niimi et al. 2017).

Based on previous findings, in the present work our aim is to investigate the interactions between chemical composition and the sensory perception of Sweetness, Sourness, and Astringency in apple juices. By focusing on juices rather than whole fruits, influences related to the mechanical properties of the apple flesh are minimized, allowing chemical contributions to sensory perception to be more prominent. We adopted a data‐driven approach integrating descriptive sensory analysis with compositional data to elucidate both bivariate and multivariate relationships among sugars, organic acids, polyphenols, VOCs, and perceived sensory attributes.

## 2 Materials and Methods

### 2.1 Apple and Juice Samples

In this study we considered thirty batches of apples sampled from the apple biodiversity collection of FEM (Fondazione Edmund Mach, San Michele all'Adige, Trento, Italy), including wild Malus species, old varieties, and selections from the FEM breeding program. Each batch consisted of one crate containing about 25–30 kg of apples. In addition, commercial varieties, namely the cultivars Granny Smith, Fuji, Golden Delicious, and Rosy Glow (Pink Lady), were included in the study as reference varieties (Supplementary Table S1). The selection criterion aimed to represent a broad range of apple phenotypic qualities, such as color, compositional characteristics, and sensory profile.

Batches of fruit were picked at commercial harvest and subsequently stored under refrigerated conditions (2°C, 98% RH) and in a normal atmosphere for 2 months.

At the end of the refrigerated storage, fruits were kept at room temperature for 24 h prior to analyses and juice production.

#### 2.1.1 Apple Fruit Characterization

For each cultivar, measurements were taken on 20 fruits selected from each crate. The selected fruits, without any visible damage, were homogeneous in mass and degree of ripeness. The following measurements were taken: the dry refractometric residue (°Brix) with a digital refractometer (Atago, PAL‐1), the Index of Absorbance Difference (IAD) (DA‐meter, Turoni s.r.l., Forlì, Italy), and the starch content estimated through the iodine test.

#### 2.1.2 Juice Extraction

For each cultivar, 5 kg of apples, previously water‐rinsed, dried, and sliced, were used for the juice production with a juice extractor (Panasonic MJ‐L500). The juice was then filtered by using a 0.7 mm strainer, and 10 mL were collected for chemical analyses, while the remaining mass was spiked with ascorbic acid (3 g/L) to improve stability until the sensory analysis was performed. The juice was poured into 250 mL bottles maintained under refrigerated conditions (3 ± 1°C) for a maximum of 36 h until high‐pressure stabilization (5000 bar for 5 s), carried out in an industrial plant (MacèFruit, Terre del Reno (FE), Italy).

The stabilized juices were stored at −20°C until the day before analysis.

### 2.2 Chemical Analyses

The chemical analyses of the juices were performed on stabilized samples that had been thawed at 4°C overnight.

#### 2.2.1 Sugars, Sorbitol, and Organic Acids

Glucose (g/L), fructose (g/L), sucrose (g/L), xylose (g/L), and sorbitol (g/L) contents were assessed by using high‐pressure capillary ion chromatography (Dionex ICS‐5000, Thermo Fisher Scientific, Waltham, MA, USA) equipped with pulsed amperometric detection (HPLC‐PAD).

Five µL of sample were injected into a Dionex UltiMate 3000 (Thermo Fisher Scientific, Waltham, MA, USA) with a Dionex CarboPac PA200 column (3 × 250 mm). As a mobile phase, a 3 step isocratic solvent system was considered (A, NaOH 1 M; B, deionized water; C, NaOH 30 mM) with a 0.3 mL min^−1^ flow rate, except for the time interval 9.5–19 min, characterized by a 0.4 mL min^−1^ flow rate: initial conditions of B (97%) + C (3%) for 13 min; then A (10%) + B (90%) for 3 min; and B (97%) + C (3%) for 5 min. Concerning calibrations, solutions of the analyzed sugars were prepared individually in deionized water and working solutions (coefficient of variation < 2% except for sucrose = 4%). Total sugar content value (g/L) was obtained in terms of the sum of glucose, fructose, sucrose, and xylose.

Malic (g/L) and ascorbic (g/L) acids contents were quantified by means of an Ultra High Performance Liquid Chromatography unit (ThermoScientific Ultimate 3000RS, Thermo Fisher Scientific, Waltham, MA, USA) coupled with an electrospray ionisation (ESI) Q ExactiveTM Hybrid Quadrupole‐Orbitrap Mass Spectrometer (Thermo Fisher Scientific, Waltham, MA, USA), by considering an injection of 20 µL of sample solution and a negative ionization mode. An analytical column (Rezex ROA Organic acid H^+^; 30 cm × 7.8 mm, 8 µm, Phenomenex, Torrance, CA) was used to separate malic and ascorbic acids. The following other operative conditions were taken into consideration: 0.8 mL min^−1^ for the mobile phase (water with 0.5% of formic acid) flow rate; 50°C for the column temperature; sheath gas flow rate of 40 arbitrary units; sweep gas flow rate of 0 arbitrary units; capillary voltage of 3500 V; capillary temperature of 350°C; and gas temperature of 300°C for ESI. Full scans (scan range 100–1500 m/z) were used for data collection, and the malic and ascorbic acid contents were conducted, respectively, at 133.01425 and 175.02481 m/z.

#### 2.2.2 Polyphenols

From each sample, an aliquot of 1 g of juice was extracted in 10 mL of a solution of methanol/water (2/1). The assessment of polyphenols was conducted with a UPLC/QqQ‐MS/MS and the samples were separated in a Waters Acquity column, kept at 40°C with water for the mobile phase A and acetonitrile containing 0.1% formic acid for the mobile phase B with a flow of 0.4 mL/min (gradient profile: 0 min, 5% B; from 0 to 3 min, linear gradient to 20% B; from 3 to 4.3 min, isocratic 20%; from 4.3 to 9 min, linear gradient 45% B; from 9 to 11 min, linear gradient 100%; from 11 to 13 min, wash at 100% B; from 13.01 to 15 min, back to the initial condition at 5% B) (Vrhovsek et al. 2012).

The separation of the anthocyanins was performed at 40°C in 30 min (linear gradients starting at 10% B, to 30% B in 10 min, to 40% B in 7 min, to 51.2% B in 4 min, to 64% B in 5 min, and to 90% B in 4 min) with an HPLC‐MS analysis (mobile phase A: 5% formic acid in H_2_O (A); mobile phase B: 5% formic acid in methanol). Prior to each analysis, the column was equilibrated for 7 min with a 0.2 mL/min flow rate and 5 µmL volume injection. The UV‐vis spectra were acquired from 230 to 600 nm with detection at 520 nm (Vrhovsek et al. 2004).

#### 2.2.3 Volatile Organic Compounds

Volatile organic compounds (VOCs) in the headspace of apple juices were collected by means of the Solid Phase Micro‐Extraction (SPME) technique according to the following procedure: 5 mL of juice was introduced into a 22 mL vial and spiked with 2‐octanol (internal standard for semi‐quantitative purposes), and headspace VOCs were extracted and concentrated on a 2‐cm SPME fiber coated with divinylbenzene/carboxen/polydimethylsiloxane (50/30 µm, DBV/CAR/PDMS, Supelco, Bellefonte, PA, USA) for 30 min, after 10 min of equilibration at 40°C. VOCs were then desorbed in the injector port of a GC (at 250°C). The GC was interfaced with a mass detector operating in electron impact ionization mode (EI, internal ionization source; 70 eV) with a scan range from m/z 35 to 300 (GC Clarus 500, PerkinElmer, Norwalk CT, USA). An HP‐Innowax fused‐silica capillary column (30 m, 0.32 mm ID, 0.5 µm film thickness; Agilent Technologies, Palo Alto, CA, USA) with He as carrier gas (constant column flow rate of 1.5 mL/min) was used for achieving the separation according to the following temperature steps: 1) 40°C for 3 min; 2) 40–220°C at 4°C/min; 3) 220°C for 1 min; 4) 220–250 at 10°C/min; 5) 250°C for 1 min.

Peaks were identified by mass spectral matching against the NIST Standard Reference Database (NIST2017) and comparison of linear retention indices (LRIs) and were confirmed, when available, by injection of authentic compounds. For the calculation of LRI, a series of n‐alkanes (C7–C30 from Supelco) was injected under the same chromatographic conditions. The complete list of identified VOCs is reported in Supplementary Table S2.

VOCs not identified in more than 40% of the samples were removed from the dataset used for statistical analyses. This threshold was selected a priori as a compromise between the retention of compounds potentially related to cultivar‐dependent aroma differences and the exclusion of highly sparse variables, which could lead to unstable correlation coefficients and feature‐importance estimates, particularly in a dataset composed of 30 samples. For the retained VOCs, non‐detected values were replaced with zero values.

### 2.3 Sensory Analysis

#### 2.3.1 Panel

The sensory evaluation was conducted with a panel of 14 trained assessors (57% female, mean age = 40.1). The panel was highly trained, with several years of experience in conducting quantitative descriptive analysis of apples (Corollaro et al. 2013).

All procedures involving participants were conducted in accordance with the principles of the Declaration of Helsinki. Sensory evaluations were recorded using alphanumeric codes to ensure participant confidentiality (pseudonymization). Personal identification data (name, age, gender, and contact details) were collected solely for panel administration and scheduling purposes. These personal records were handled exclusively by authorized staff and kept strictly separate from the sensory dataset, in compliance with applicable data protection regulations at the time of the study. Participation was voluntary, and no monetary compensation was offered.

#### 2.3.2 Samples

The samples utilized in this study are detailed in Section 2.1. The juice samples were stored frozen at −20°C in 250 mL plastic bottles following microbiological stabilization. One day prior to evaluation, the required samples were transferred to a refrigerator to thaw at 4°C overnight. For the sensory sessions, aliquots of 25 mL were served at 15°C in 80 mL black plastic cups, each labelled with a unique three‐digit random code. The cups were presented to the panelists covered with a plastic lid.

#### 2.3.3 Procedure

The study comprised eight sessions in total: three dedicated to panel training (Sessions 1–3) and five to the formal evaluation of the apple juice samples (Sessions 4–8). As the scope of the present analysis is confined to the data from the intensity scales, only a brief overview of the Pivot Profile method (Thuillier et al. 2015) is provided.

The training protocol consisted of three sessions. In Session 1, panelists were re‐familiarized with sensory attributes for which they had been previously trained in other research projects (e.g., Aprea et al. 2017; Charles et al. 2017; Corollaro et al. 2013). This session utilized reference standards developed in prior studies on apples to recall specific odors, tastes, and sensations. Specifically, the panel evaluated eleven medium‐intensity odor standards (pineapple, banana, kiwi, lemon, melon, pear, grass, hay, anise, vanilla, and honey) (Charles et al. 2017). Additionally, three taste standards (sweet, bitter, and sour) and one trigeminal sensation standard (Astringency) were presented at both low and high intensities (Corollaro et al. 2013). In Session 2, the panel engaged in lexicon development, generating sensory terms using the Napping method (Lê et al. 2023) to describe the odor and flavor attributes of a representative subset of the apple juices (*n* = 10). Session 3, served to introduce the formal evaluation procedure, which was practiced using three samples (H. Shelley, Golden Delicious, and Fuji) presented in a randomized order. The procedure for Session 3, which was subsequently used in all formal evaluation sessions, involved three stages: (i) Pivot Profile of odor, (ii) Pivot Profile of flavor, and (iii) intensity ratings of basic tastes. In the Pivot Profile stages, panelists were instructed to compare each sample to a designated ‘Pivot’ juice. For the odor profile, they smelled each sample and recorded the attributes that were perceived as either more or less intense compared to the Pivot, using the lexicon generated in Session 2. A mandatory 30‐second break was observed between samples. For the flavor profile, panelists received a new tray with the same set of samples and repeated the comparative process by tasting. Following the Pivot Profile evaluation for each sample, panelists rated the intensity of Sweetness, Sourness, and Astringency attributes on a 100‐point unstructured line scale, anchored with “extremely weak” at 0 and “extremely strong” at 100. During the tasting phases, panelists were instructed to use a drinking straw and not to remove the lid. A 60‐second break, during which they rinsed their mouths with water, was required between each sample tasting.

Following the successful completion of training, the panel proceeded to five formal evaluation sessions (Sessions 4–8). In each session, six juice samples were presented in a randomized order and evaluated using the protocol established in Session 3. The assignment of samples to each session was balanced to ensure variability in total sugar content and color across the sessions. Each sample was presented as a single replicate to limit panelist fatigue and to remain within the product quantities available for the study.

All formal evaluations (Sessions 4–8) and the final training (Session 3) were conducted in individual sensory booths compliant with EN ISO 8589:2010 and equipped with red lighting to mask visual differences between samples. Data were recorded electronically using Fizz sensory analysis software (version 2.51, Biosystèmes, Couternon, France).

### 2.4 Data Analysis

The consistency and discriminant ability of the assessors for the attributes of Astringency, Sourness, and Sweetness were evaluated using a *p*‐value vs. Mean Square Error (MSE) plot (Næs et al. 2010). In this plot, the *p*‐value, derived from an ANOVA for each assessor and attribute, indicates the assessor's ability to discriminate among the samples. The MSE, in contrast, represents the repeatability of an assessor's evaluations. An effective assessor is characterized by a combination of a low *p*‐value (typically < 0.05) and a low MSE value (e.g., < 400). Panel agreement was subsequently evaluated using Tucker‐1 correlation loading plots (Næs et al. 2010). For a well‐trained panel, the correlation loadings for a given attribute should be situated near the outer ellipse (representing 100% explained variance), with individual assessors plotted near one another. Both analyses were performed using samples H. Shelley, Golden Delicious, and Fuji from pre‐test data (training session 3) and from final test. Analyses were performed in R with scripts derived from the PanelCheck V1.4.2 software (Nofima Mat, Technical University of Denmark and University of Copenhagen).

All the collected data on apple fruits and juices were described in terms of mean, standard deviation, minimum, and maximum values. Linear (*r*) or monotonic (ρ, if *p* < 0.05 for normality assumption) correlation coefficients were calculated for bivariate correlation analysis between fruit characteristics (dry refractometric residue, Index of Absorbance Difference, and starch content), chemical composition (glucose, fructose, sucrose, xylose, sorbitol, malic and ascorbic acids), and sensory attributes (sweetness, sourness, and astringency) of juices. Linear (*r*) or monotonic (ρ, if *p* < 0.05 for normality assumption) relationship between sensory attributes (Sweetness, Sourness, and Astringency) and the identified polyphenols, anthocyanins, dihydrochalcones, and VOCs were also discussed.

The significance of the above‐described correlation analyses was tested according to a significant level of α of 0.05, and data independence was explored within each feature through the analysis of the autocorrelation function plot. In addition, the Benjamini–Hochberg procedure was used as a method to control, in multiple hypothesis testing, the expected proportion of false discoveries.

The sample size considered in this work (30 apple batches) gives good power (≈ 0.83) to detect moderate correlations (e.g., *r* = 0.5) between chemical measures and sensory attributes while a reduced sensitivity to smaller effects (*r* < 0.5) is acknowledged. Furthermore, this sample size satisfies the common statistical criterion (*n* ≥ 30) for approximating normality in mean estimates, providing a robust basis for parametric analyses (e.g., ANOVA and regression).

Support Vector Machine regression (SVMR) models (algorithm: libsvm; SVM type: epsilon‐SVR; SVM kernel type: radial basis function) were explored with the aim of identifying the importance of the features describing the dependent variables sweetness, sourness, and astringency (Solo, Version 9.5.0.0 (1), Eigenvector Research). Independent variables were preprocessed by performing the “group scaling” procedure (three classes according to the chemical components, polyphenol and VOC groups of variables), based on standard deviations with mean centering. Concerning VOCs, non‐detected values were replaced with “0” values. SVMR tool can be considered one of the machine learning tools particularly useful also in the case of non‐linear relationships when the number of independent variables is greater than the number of samples (Bishop 2006).

For each dependent variable, an explorative SVMR model was built starting from a matrix containing all 139 independent features (dry refractometric residue, Index of Absorbance Difference, starch content, glucose, fructose, sucrose, xylose, sorbitol, malic acid, identified polyphenols, anthocyanins, dihydrochalcones, and VOCs).

SVM regression model hyperparameters (cost: 0.001, 0.003162278, 0.01, 0.03162278, 0.1, 0.3162278, 1, 3.162278, 10, 31.62278, 100; epsilon: 0.01, 0.1, 1; gamma: 1e‐06, 3.16228e‐06, 1e‐05, 3.16228e‐05, 0.0001, 0.000316228, 0.001, 0.00316228, 0.01, 0.0316228, 0.1) were optimized through grid search method used with the aim of investigating all the combinations. The predictive ability was tested by using the random subsets cross‐validation method (numbers of data splits = 8; numbers of iterations = 6). Cross validations were repeated 10 times, and results are shown in terms of means and standard deviation.

Features describing each sensory attribute were then selected through the analysis of correlation coefficients included in the 60th superior percentile. Starting from this selection, new SVMR models were built, and, in order to better understand how each variable in the dataset contributes to samples’ prediction, Shapley Values were calculated and discussed. Shapley values, introduced by Shapley in 1953 as a tool to distribute the value of a game, the payout, among its players according to a cooperative game theory (Shapley 1953), are also used in the machine learning context to define feature importance in classification and prediction models (Štrumbelj and Kononenko 2010; Sundararajan and Najmi 2020). In the machine learning applications, the players are represented by the features, and a value function ν is created with the aim of extracting Shapley Values consistent with the estimation. In simple words, the values can be considered a weighted mean of the differences in the value of the “game” (prediction) caused by the participation of the “player” (feature) in all possible coalitions (Aas et al. 2021; Borgonovo et al. 2024). Shapley values were considered to estimate how each feature contributes to the model's output (highest Shapley values indicate the most important dataset features for each sensory attribute). It should be noted that, in the present work, correlation coefficients and Shapley values were considered as associative and model‐based indicators of contribution, rather than as evidence of independent causal effects.

## 3 Results and Discussion

### 3.1 Panel Performance

Analysis of the data from the training samples (H. Shelley, Golden Delicious, and Fuji) across the third training session (pre‐test) and the final test revealed that the majority of the assessors fell within the optimal region of the *p*‐value vs. MSE plot (p < 0.05; MSE < 400) for the attributes of Astringency (64.3% of assessors), Sourness (85.7%), and Sweetness (85.7%) (Figure S1). Additionally, visual inspection of the Tucker‐1 plots from both sessions confirmed that all assessors were positioned between the 50% and 100% explained variance ellipses for all attributes (Figure S2). The distinct clustering of the panelists in these plots further indicates a high degree of consensus in their application of the Astringency, Sourness, and Sweetness descriptors. These results collectively demonstrate that the panel exhibits robust evaluation performance for the three attributes under study.

### 3.2 Apple Fruits Characteristics, Juice Composition, and Sensory Analyses

Main parameters measured starting from both apple fruits and apple juices are summarized in terms of mean, standard deviation (SD), minimum (Min), and maximum values (Max) for the 30 considered batches (Table 1). In Supplementary Table S3 we included a more exhaustive table also showing a descriptive summary for the three sensory attributes sweetness, sourness, and astringency assessed on the produced juices on a 0–100 mm scale and linear (r) or monotonic (ρ, if p < 0.05 for normality assumption) relationships with main attributes from both apple fruits and apple juices in addition to p‐adjusted values according to the Benjamini–Hochberg procedure.

**TABLE 1:** Apple fruits and apple juices characteristics and sensory attribute scores.

	Mean	SD	Min	Max
*Apple fruit*				
*I*_AD_	1.04	0.48	0.04	1.84
Starch (Iodine test)	8.5	1.8	3.0	10.0
DRR(°Brix)	12.2	1.4	10.1	16.2
*Apple juice*				
Glucose (g/L)	24.1	5.1	17.0	37.0
Fructose (g/L)	62.1	10.6	44.0	92.0
Sucrose (g/L)	34.2	14.2	3.4	68.0
Xylose (g/L)	0.55	0.27	0.28	1.34
Sorbitol (g/L)	5.4	2.7	1.7	13.6
Malic acid (g/L)	8.02	3.70	3.25	19.40
Ascorbic acid (g/L)	1.22	0.54	0.34	2.98
Sweetness (0–100 scale)	34.5	17.8	4.2	66.8
Sourness (0–100 scale)	52.1	25.2	11.1	92.5
Astringency (0–100 scale)	44.7	17.4	11.5	77.7

Abbreviations: DRR (°Brix) dry refractometric residue; IAD, Index of Absorbance Difference; SD, Standard deviation.

As can be appreciated, the chosen batches of apple fruits are characterized by a valuable range of variability in terms of the analyzed three maturity indices. These ranges reflect the apple variability harvested at commercial maturity and stored for two months under refrigerated conditions (Aprea et al. 2017) (Sjöstrand et al. 2024).

Passing to apple juice and concerning total sugar content, the analyzed batches were characterized by a content ranging from 86.2 (g/L) to 175.0 (g/L) with an average value of 121.0 (g/L). By considering mean values, fructose (62.1 g/L) and sucrose (34.2 g/L) appeared to be the most representative sugars, followed by glucose (24.1 g/L) and the main sugar alcohol sorbitol (5.4 g/L), as also evidenced by Eisele and Drake (2005) in apple juices.

For organic acid content, mean values of 8.02 g/L (maximum value of 19.40 g/L) and 1.22 g/L (maximum value of 2.98 g/L) were observed for malic acid, the main organic acid present in apples, and for ascorbic acid content, added to the juice samples. The malic acid sample values are representative of apples harvested at the commercial maturity stage (Ma et al. 2015; Wu et al. 2007).

Passing to sensory attributes, fruit juice samples were characterized by a considerable variability in terms of sweetness (from 4.2 to 66.8), sourness (from 11.1 to 92.5), and astringency (from 11.5 to 77.7). As can be seen from the mean values, the samples analyzed were sourer (52.1) and more astringent (44.7) than sweet (34.5).

As shown in Table S3, in terms of linear or monotonic correlation coefficients, for sweetness, there is a positive significant correlation with sucrose (0.479) and a negative significant correlation with malic acid (−0.634). Similar results were obtained by Ma et al. (2015) for cultivated and wild apples. The dry refractometric residue (°Brix) of apple fruit showed to be not significantly related to sweetness (0.349).

Sourness and astringency are positively and significantly related to malic acid (0.880 and 0.792, respectively), and, only for astringency, although weak, a positive and significant correlation with sorbitol content (0.364) can also be appreciated. As reported in the literature, acid compounds, in addition to phenolic compounds, salts of multivalent metallic cations, and dehydrating agents, are responsible for the drying, puckering, and shrinking sensation in the oral cavity (Bajec and Pickering 2008).

### 3.3 Polyphenols and Volatile Organic Compounds (VOCs), and Interaction With Sensory Attributes

Mean, standard deviation (SD), minimum (Min), and maximum values (Max) for the 30 batches considered were summarized in Table 2 for a list of identified polyphenols, characterizing more than 40% of the samples. Anthocyanins were not identified in more than 40% of the samples. By analyzing significant linear or monotonic coefficients calculated for bivariate relationships with sensory attributes (Table S4), astringency appeared to slightly related, in a positive and significant way, to both polyphenols, chlorogenic acid (0.383), procyanidin B2 + B4 (as B2) (0.367), and quercetin‐3‐O‐glucoside (Qu3gluc) + quercetin‐3‐O‐galactoside (Qu3gal) (0.462), and to dihydrochalcones phlorizin (Pz, 0.372), and phloretin‐2‐O‐xyloglucoside (Ptxlgluc) (0.502). These compounds are reported to be responsible for astringency of different fruit and vegetable non‐alcoholic beverages (Chen et al. 2022; Shanker et al. 2024; Verdu et al. 2014).

**TABLE 2:** Polyphenols in apple juices.

	Mean	SD	Min	Max
*Polyphenols* (𝝻g/g)				
4‐Aminobenzoic acid	0.039	0.023	0.010	0.098
Vanillin	0.044	0.027	0.011	0.119
Vanillic acid	0.038	0.149	0.000	0.814
Caffeic acid	0.16	0.39	0.00	1.64
Neochlorogenic acid	0.34	0.57	0.04	3.23
Cryptochlorogenic acid	8.9	11.8	0.81	65.8
Chlorogenic acid	122	88.3	10.8	299
Catechin	5.4	6.4	0.0	29.3
Epicatechin	31.5	32.5	0.5	170
Procyanidin B1	27.4	27.5	0.0	107
Procyanidin B2 + B4 (as B2)	119	146	0.12	685
Quercetin‐3‐Rha	0.62	1.09	0.01	5.73
Quercetin‐3‐Glc + Quercetin‐3‐Gal (as Que‐3‐Glc)	2.1	3.2	0.0	17.1
*Dihydrochalcones* (𝝻g/g)				
Phlorizin	1.9	5.6	0.0	30.3
Phloretin Xylo Glc	6.3	10.5	0.4	56.1

Abbreviations: Ga, galactoside; Glc, glucoside; Que, quercetin; Rha, rhamninoside; Xylo Glc, xylo‐glucoside.

A general negative influence on sweet taste emerges with the same strength for the same polyphenols (chlorogenic acid, procyanidin B2 + B4, Qu3gluc + Qu3gal) and the dihydrochalcones Ptxylgluc compounds. This behavior could be mainly attributed to the strong indirect relationships (*r* = −0.803) that can be observed between sweetness and astringency sensory attributes. Similarly, the correlations between sourness and Qu3gluc + Qu3gal (*r* = 0.378) and Ptxylgluc (*r* = 0.432) could be explained through the strong direct relationships observed between sourness and astringency (*r* = 0.873).

Table 3 reports the mean, standard deviation (SD), minimum (Min), and maximum values (Max) for the 30 apple batches considered. A list of 52 VOCs (from a total of 117 identified VOCs in more than 40% of the samples), were significantly correlated (*p* < 0.05) (with linear or monotonic relationships coefficients) with sensory attributes, is shown in Table S5.

**TABLE 3:** Volatile organic compounds significantly correlated (α = 0.05) with sensory attributes.

Volatile compounds (µg/L)	Mean	SD	Min	Max
***Esters***				
Propyl acetate	217	403	n.d.	1502
Isobutyl acetate	241	296	n.d.	1094
Propyl propanoate	119	385	n.d.	2041
Butyl acetate	9629	11228	72.7	35572
Isoamyl acetate	12496	15499	27.6	50459
Propyl butanoate	1288	3505	n.d.	17190
Propyl‐2‐methyl butanoate	235	570	n.d.	2544
Amyl acetate	2065	2858	8.88	11596
Butyl butanoate	4941	5267	114	18151
Butyl‐2‐methyl butanoate	589	903	3.53	3533
Isoamyl butanoate	45.2	62.9	n.d.	245
Hexyl acetate	22900	33730	138	1E+05
Propyl hexanoate	154	412	n.d.	1977
(Z)‐3‐Hexen‐1‐ol acetate	399	848	n.d.	3972
(E)‐2‐hexenyl acetate	79.9	104	1.13	397
Butyl hexanoate	134	166	n.d.	616
Hexyl butanoate	1989	3074	9.95	13008
Hexyl hexanoate	168	227	11.1	1035
Butyl‐3‐hydroxybutanoate	22	19.5	n.d.	77.1
Hexyl (E)‐2‐methyl‐2‐butenoate	4.18	7.58	n.d.	38.7
2‐Phenethyl acetate	11.4	29.5	n.d.	162
(E)‐3‐Hexenyl acetate	18.1	25.2	n.d.	84.6
Isopropyl myristate	10.7	3.69	4.98	19.6
1‐Methoxyethyl benzoate	9.68	1.99	6.5	15.1
***Aldehydes***				
Butanal	31.3	44.5	n.d.	200
Hexanal	5023	3584	192	13013
2‐Methyl‐2‐butenal	14.2	35.3	n.d.	194
(E)‐2‐Hexenal	5469	4961	392	19516
2,4‐Hexadienal (E,E)	28	28.6	3.9	141
2,4‐Hexadienal (E,Z)	64.7	64.3	8.49	287
2,4‐Dimethyl benzaldehyde	17.9	4.98	9.16	29.4
***Alcohol***				
2‐Heptanol	48.2	105	n.d.	415
Hexanol	22179	14435	3395	54882
Nonanol	30.8	12.3	13.2	62.7
2‐Nonanol	47	106	n.d.	463
1‐Butanol	2554	2858	129	12263
Pentanol	261	175	47.4	748
1‐Dodecanol	28.3	8.22	14.7	40.9
***Ketones***				
2‐Octanone	515	55.3	377	630
***Acids***				
2‐Methylbutanoic acid	31.6	79.2	n.d.	405
Pentanoic acid	8.39	10.8	n.d.	33.6
Hexanoic acid	62.6	32	26.1	143
Octanoic acid	36.4	22.4	11.6	133
Dodecanoic acid	27.4	8.21	16.3	50.2
***Terpenes and derivatives***				
α‐Terpineol	10.7	6.8	4.75	35.8
(Z,E) ‐α‐Farnesene	16.1	14.1	2.47	63.9
p‐Allylanisole	286	553	12.1	2028
Anethol	31.2	26.6	7.82	110
β‐Damascenone	64.7	46.8	4.43	233
***Others***				
1‐Hexadecene	225	57.4	138	370
5‐Ethyldihydro‐2(3H)‐furanone	6.85	6.42	n.d.	25.5
Benzothiazole	4.48	2.34	1.71	11.0

Abbreviations: n.d., not detected

In general, the major group of significantly correlated VOCs is represented by esters, evidenced also in a previous work (Aprea et al. 2017). Within the identified VOCs, 8 esters are characterized by positive coefficients higher than 0.500 for sweetness (propyl acetate, isobutyl acetate, butyl acetate, amyl acetate, butyl‐2‐methyl butanoate, hexyl acetate, butyl‐3‐hydroxybutanoate, and 1‐methoxyethyl benzoate). Particularly, acetate esters are associated with fruity odor and flavor that intensify according to ripening (Aprea et al. 2017; Li et al. 2025; Pino and Febles 2013) and are also considered sweetness‐associated compounds for sucrose solutions (Xiao et al. 2024). As is known, esters are the main VOCs characterizing apple juices, followed by aldehydes (Guo et al. 2020). Concerning aldehydes, hexanal, (E)‐2‐hexenal, (E,Z)‐2,4‐hexadienal, and 2,4‐dimethyl benzaldehyde show positive coefficients higher than 0.500 for sweetness. Within significant VOCs, hexanal appears to be, in terms of strength, the most associated with the perception of sweetness in apple juice (0.704). Hexanal, a natural by‐product of lipid peroxidation (Aprea et al. 2018), is highly dependent on cultivar and imparts, in addition to the related (E)‐2‐hexenal, 2,4‐hexadienals, green and cucumber‐like notes (Schieberle et al. 1990; Vallone et al. 2013). According to research conducted by Charles et al. (Charles et al. 2017), apple sweet odor perception can be highlighted following the addition of (Z)‐3‐hexen‐1‐ol, reported as green/cut grass for the vegetal category. Based on the observation of the latter work, we speculate that hexanal indirectly contributes to the sweetness perception by contributing to the fresh green odor. Regarding (E)‐2‐hexenal, that has been demonstrated that this compound activates human TAS1R2/TAS1R3 sweet taste receptors in vitro (Horie et al. 2025). The same study reports that in a small human sensory test with nose clips, 5 mM (E)‐2‐hexenal was rated sweeter than water and not significantly different from 50 mM sucrose.

In addition, alcohols and other VOCs significantly correlate with tested juice sweetness. Among them α‐terpineol, associated with a pleasant floral odor, is a naturally occurring volatile component in apples and apple juices (Marsol‐Vall et al. 2017).

It is worth mentioning that these compounds influence sweetness perception mainly through odor–taste interactions (Small and Prescott 2005) rather than by direct effect. This is a well‐documented example of odor–taste synergy, where congruent aromas (those typically experienced with sweet foods) amplify sweetness perception. Their influence is thus largely synergistic, shaping how sweetness is perceived rather than reflecting actual sweeteners’ content.

Concerning sourness and astringency, the calculated significant correlations, all negative, appeared to be mainly related to the indirect relation with sweetness perception. Positive correlations can be observed for 2‐methyl‐2‐butenal (sourness and astringency), 2‐octanone (sourness), and β‐damascenone (sourness and astringency). The odors associated with 2‐methyl‐2‐butenal are commonly pungent, green, ethereal, and sharp and penetrating (https://www.thegoodscentscompany.com), congruent with both sourness and astringency; the 2‐octanone odor is described as earthy; and β‐damascenone is recognized as a potential aroma‐enhancing compound. β‐damascenone has an extremely low odor threshold, and its odor quality is concentration‐ and matrix‐dependent, with trace levels often enhancing floral/fruity character and higher levels tending toward cooked fruit, honey, and tobacco (Tomasino and Bolman 2021). The positive correlation between β‐damascenone and both sourness and astringency is consistent with the shift from floral to tobacco odor.

### 3.4 Studying the Interaction Between Fruit Maturity, Juice Characteristics, and Sensory Attributes

With the aim of better identifying the importance of the features describing the three analyzed sensory attributes in an integrated and supervised way, three SVMR models were built starting from all independent features (*n* = 137, dry refractometric residue, glucose, fructose, sucrose, xylose, sorbitol, malic acid, identified polyphenols, anthocyanins, dihydrochalcones, and VOCs). Examples of plots of predicted versus observed values from SVMR for sweetness, sourness and astringency are reported in the Supplementary Material (Figure S3).

The importance of the features describing the three sensory attributes was evaluated by analyzing the extracted Shapley Values obtained from SVMR models built from the reduced number of features selected through the analysis of correlation coefficients included in the 60th percentile (sweetness = 55 independent variables; sourness = 56 independent variables; astringency = 55 independent variables).

These values provide a general explanation of the model by aggregating the Shapley Value matrix across all samples (the entire calibration data set) and for every variable. Accordingly, for each sensory attribute, Table 4 reports the independent variables whose absolute Shapley values fell within the 75th percentile, together with the corresponding standard deviations obtained across 10 cross‐validation replications and the direction of their contribution. Beewarm plots for Shapley Values for sweetness, sourness, and astringency models are reported in the Supplementary Material (Figures S4–S6).

**TABLE 4:** Shapley Values for sensory attributes SVM regression models.

Sensory attribute	Variable	Shapley Value*	Direction of contribution
Sweetness	Malic acid	6.271 (0.568)	−
	Procyanidin B2 + B4 (as B2)	4.632 (0.645)	−
	Sucrose	4.268 (0.355)	+
	Isoamyl acetate	2.015 (0.142)	+
	Hexyl acetate	1.886 (0.176)	+
	(E)‐2‐Hexenal	1.584 (0.281)	+
	Hexyl butanoate	1.120 (0.239)	+
	Epicatechin	1.034 (0.389)	−
	Hexanal	1.008 (0.221)	+
	Hexanol	0.985 (0.448)	+
	Propyl butanoate	0.680 (0.122)	+
	Butyl butanoate	0.593 (0.569)	+
	Butyl acetate	0.569 (0.207)	+
	DRR (°Brix)	0.377 (0.154)	+
Sourness	Malic acid	18.493 (0.797)	+
	Butyl acetate	1.775 (0.102)	−
	(E)‐2‐Hexenal	1.577 (0.236)	−
	Sorbitol	1.511 (0.164)	+
	Hexanol	1.133 (0.348)	−
	Procyanidin B2 + B4 (as B2)	1.009 (0.810)	+
	Chlorogenic acid	0.905 (0.543)	+
	Hexyl acetate	0.851 (0.359)	−
	Propyl butanoate	0.814 (0.284)	−
	1‐Butanol	0.511 (0.440)	−
	Hexyl butanoate	0.507 (0.362)	−
	Butyl butanoate	0.492 (0.317)	−
	Benzaldehyde	0.369 (0.256)	+
	Butyl propanoate	0.325 (0.097)	−
Astringency	Malic acid	7.344 (0.775)	+
	Procyanidin B2 + B4 (as B2)	2.530 (1.071)	+
	Hexyl acetate	2.497 (1.028)	−
	Butyl acetate	2.442 (0.434)	−
	Hexanol	2.427 (0.419)	−
	(E)‐2‐Hexenal	1.004 (0.059)	−
	Chlorogenic acid	0.901 (0.125)	+
	Sorbitol	0.894 (0.230)	+
	Butyl butanoate	0.780 (0.322)	−
	Epicatechin	0.603 (0.042)	+
	Hexanal	0.514 (0.072)	−
	2‐Methylbutanoic acid	0.293 (0.186)	−
	2‐Methyl‐1‐butanol	0.236 (0.075)	−
	Amyl acetate	0.102 (0.065)	−

^*^Means and standard deviation, in parentheses, of 10 cross validation replications (absolute values included in the 75^th^ percentile).

SVMR models, validated with the random subsets cross‐validation method (number of data splits = 8; numbers of iterations = 6), showed the following characteristics (average of 10 cross‐validation replications): sweetness (R^2^CV = 0.615 ± 0.034, RMSCV = 10.9 ± 0.476), sourness (R^2^CV = 0.746 ± 0.072, RMSCV = 12.3 ± 1.5), and astringency (R^2^CV = 0.620 ± 0.056, RMSCV = 10.3 ± 1.2).

The following SVM regression model hyperparameters were optimized by software: sweetness (cost = 100; epsilon = 0.1; gamma = 0.1); sourness (cost = 100; epsilon = 0.1; gamma = 0.03); astringency (cost = 100; epsilon = 0.1; gamma = 0.01). The Shapley values reported in Table 4 should be interpreted as descriptive indicators of feature contribution in the final SVMR models, rather than as inferential estimates of variable‐ranking stability. Accordingly, the relative order of variables with similar or low Shapley values should be interpreted with caution.

It should be considered that, due to the collinearity among some predictors, Shapley values indicate the contribution of each variable to model prediction and should not be interpreted as independent causal effects of single compounds.

In general, for all sensory attributes, the analysis of the reported highest Shapley Values confirms what was observed after bivariate correlation in terms of how each feature contributes to the prediction of a single sensory attribute. For sweetness, the main role of sucrose (4.268, positive direction of contribution) as well as the indirect relationships with malic acid (6.271, negative direction of contribution) and procyanidin B2 + B4 (as B2) (4.632, negative direction of contribution). Even if with a lower magnitude, the contribution of (E)‐2‐Hexenal (1.584, positive direction) and hexanal (1.008, positive direction) content is confirmed. Butyl and acetate esters also show positive contributions to sweetness, confirming once again the role of the esters associated with fruity odors as sweetness enhancers (Table 4).

Malic acid had the highest positive weight on both sourness (Shapley Values of 18.493), as expected, and astringency (Shapley Values of 7.344). The contribution of malic acid to astringency prediction, we speculated, could be associated with the fact that sourness and astringency in apple juice are strongly correlated. Procyanidin B2 + B4 (as B2), chlorogenic acid, and epicatechin, even if with a small magnitude, are confirmed as positive contributors to astringent sensation in apple juice as well. The Shapley value for the astringency model (Table 4) shows that several esters (butyl acetate, butyl butanoate, hexyl acetate, and amyl acetate) associated with sweet/fruity flavors contribute negatively to astringency in apple juice. In a recent work isoamyl acetate (sweet/fruity flavor) was shown to moderately reduce astringency in aqueous solutions (Rollinat et al. 2025).

### 3.5 Limits of the Study

In the present study, we examined sugars, organic acids, polyphenols, VOCs, and their interactions in shaping the perceived sensory attributes of sweetness, sourness, and astringency in apple juice. These compounds were prioritized because they represent the primary chemical drivers of these sensory dimensions. We cannot, however, exclude potential contributions from other juice components. As for free amino acids, small peptides, and minerals, their relatively low concentrations in apple juice led us to assume that they are unlikely to exert a predominant influence on the sensory attributes investigated.

Even though juices were produced by a juice extractor, soluble pectin is expected to be present on the order of hundreds of mg/L (Kahl et al. 2017). In a recent study, Li and coworkers (2025) investigated how pectin content impacts the typical aroma of apple juice. Pectin reduces the release of key fruity and green VOCs, especially esters and aldehydes, thereby lowering perceived sweetness and freshness in apple juice. It can also enhance the release of some alcohols, which contributes to floral notes and a longer‐lasting finish. These effects arise from both viscosity‐driven suppression of volatility and direct molecular interactions between pectin (via D‐galacturonic acid) and aroma compounds. For these reasons, an indirect effect of pectin on the perception of sweetness, sourness, and astringency cannot be excluded. Further research is needed to elucidate how pectin may influence taste perception in apple juices.

As reported in Section 2.1.2, ascorbic acid was added at 3 g/L to all juice samples to limit oxidation and improve sample stability before sensory evaluation. Because the treatment was applied uniformly, it was not expected to explain relative differences among samples. However, ascorbic acid may have contributed to the absolute level of sourness and may have influenced volatile stability or release. Since the experimental design did not include untreated control juices, the influence of added ascorbic acid on sourness, volatile release, and overall sensory perception cannot be excluded. Future studies should include a comparison between juices with and without ascorbic acid addition to quantify this effect.

As outlined in Section 2.4, the sample size employed in this study (30 apple batches) provides adequate statistical power to detect moderate correlations. Nonetheless, although the dataset captures a degree of practical variability, it may not fully represent the broader chemical and sensory diversity present across the apple population. Future research incorporating larger and more heterogeneous sample sets, encompassing additional cultivars, harvest conditions, and storage regimes, would enhance the precision of effect‐size estimates and improve the robustness and generalizability of the resulting predictive models.

In the formal sensory evaluation, each juice was assessed as a single replicate per assessor, which prevents a complete separation of the assessor × product interaction from residual error. Future studies incorporating full within‐product replication would allow a more rigorous decomposition of panelist and product variance.

## 4 Conclusion

This study investigates the extent to which different chemical attributes contribute to the sensory perception of sweetness, sourness, and astringency in apple juice. Using a combination of classical bivariate correlation analysis along with unsupervised and supervised statistical tools, the relationships among key chemical variables and their relative importance in explaining apple juice taste were evaluated in an integrated, interactive manner.

Our findings confirm the interaction between fruity esters and compounds imparting fresh herbaceous notes, such as hexanal and (E)‐2‐hexenal, and sugars in influencing sweetness perception in apple juice, confirming our previous findings. While most previous research has primarily focused on sweetness, this study is among the first to systematically analyze the relationships between chemical composition and sourness and astringency in apple juices. Our data indicate that VOCs play a significant role in modulating not only sweetness but also, to some extent, sourness and astringency. Furthermore, quercetin and phloretin glycosides were found to be strongly associated with both sourness and astringency.

Overall, these results emphasize the multisensory and interactive nature of taste perception in apple juice, highlighting that sweetness, sourness, and astringency are influenced by a complex interplay between VOCs and non‐volatile compounds. The insights provided by this study contribute to a better understanding of how chemical composition shapes sensory experience and could inform apple breeding, juice formulation, and product optimization strategies aimed at enhancing desirable flavor attributes.

We applied Support Vector Machine regression to explore relationships between sensory scores and instrumental measurements and to generate predictive models. The analysis accounted for interactions among chemical variables and estimated the contribution of individual compounds to sweetness and, for the first time, to sourness and astringency in apple juice.

## Author Contributions

**Annachiara Berardinelli**: writing – original draft, validation, visualization, writing – review and editing. **Danny Cliceri**: investigation, methodology, validation, writing – review and editing, and formal analysis. **Isabella Endrizzi**: investigation, writing – review and editing, methodology, validation, formal analysis, and data curation. **Flavia Gasperi**: conceptualization, investigation, funding acquisition, writing – review and editing, methodology, project administration, supervision, and data curation. **Urska Vrhovsek**: investigation, writing – review and editing. **Eugenio Aprea**: conceptualization, investigation, funding acquisition, writing – original draft, writing – review and editing, methodology, formal analysis, data curation, and supervision.

## Conflicts of Interest

The authors declare no conflicts of interest.

## Supporting information




**Supplementary Material**: jfds71479‐sup‐0001‐FigureS1‐S6.docx

**Supplementary Material**: jfds71479‐sup‐0002‐TableS1‐S5.docx
