# Supplementary material for: Chemical Drivers of Sweetness, Sourness, and Astringency in Apple Juice Identified Using Support Vector Machine Regression
Source: J Food Sci. 2026 Sep 16;91(9):e71479. doi: 10.1111/1750-3841.71479 (PMC13579707; doi:10.1111/1750-3841.71479)
Supplement: Supplementary file 1 [file JFDS-91-0-s001.docx]

**Table S1. Apple accessions and cultivars included in the study.**

| **Accession** | **Flesh colour** | **Juice yield (%)** |
| --- | --- | --- |
| Baumanns Renette | yellow | 77 |
| 7.16.18 | red | 59 |
| Ribston Pepping | yellow | 84 |
| Otterson | red | 64 |
| Champion | yellow | 62 |
| 7.16.11 | red | 72 |
| Edelbohmer | yellow | 64 |
| Mela Roggia | yellow | 64 |
| Kanada Renette | white/green | 58 |
| Cavicchio | yellow | 68 |
| Tardivi | yellow | 62 |
| Fiesta | white | 66 |
| 7.16.07 | red | 69 |
| 7.15.20 | red | 67 |
| Oldengburg Red | white | 55 |
| Jonafree | yellow | 65 |
| Wagner | yellow | 53 |
| Bellefleur Record | pink | 78 |
| Golden Delicious | yellow | 62 |
| Welschbrunner | yellow | 53 |
| Malaga | white | 68 |
| H. Shelley | red | 39 |
| Verder Rosato | yellow | 58 |
| Rosmarina Rossa | yellow | 56 |
| M.J. Darking | red | 65 |
| Granny Smith | yellow | 64 |
| Fem 16 | yellow | 62 |
| Fuji | yellow | 63 |
| Fem 2 | yellow | 63 |
| Rosy Glow | white | 62 |

**Table S2. List of the identified GC-MS VOCs.**

| **VOC no.** | **Identified VOC** | **Chemical class** | **RT** | **LRI_cal_** | **LRI_nist_** |  |
| --- | --- | --- | --- | --- | --- | --- |
| 1 | Butanal | aldehyde | 2.76 | 886 | 877 | STD |
| 2 | Ethyl acetate | ester | 2.92 | 901 | 888 | STD |
| 3 | Propyl acetate | ester | 4.54 | 986 | 973 |  |
| 4 | Pentanal | aldehyde | 4.62 | 990 | 979 |  |
| 5 | Methyl butanoate | ester | 4.76 | 997 | 982 |  |
| 6 | Methyl-2-methyl butanoate | ester | 5.36 | 1022 | 1009 |  |
| 7 | Isobutyl acetate | ester | 5.43 | 1025 | 1012 |  |
| 8 | 1-Decene | hydrocarbon | 6.02 | 1049 | 1050 |  |
| 9 | Ethyl butanoate | ester | 6.05 | 1050 | 1035 |  |
| 10 | Toluene | hydrocarbon | 6.13 | 1053 | 1042 |  |
| 11 | Propyl propanoate | ester | 6.28 | 1060 | 1044 |  |
| 12 | Ethyl-2-methyl butanoate | ester | 6.45 | 1066 | 1051 |  |
| 13 | Butyl acetate | ester | 7.05 | 1091 | 1074 |  |
| 14 | Hexanal | aldehyde | 7.35 | 1102 | 1083 | STD |
| 15 | 2-methyl-2-butenal | aldehyde | 7.69 | 1112 | 1095 |  |
| 16 | 2-methyl-1-propanol | alcohol | 7.71 | 1112 | 1092 |  |
| 17 | Isoamyl acetate | ester | 8.58 | 1138 | 1122 | STD |
| 18 | Propyl butanoate | ester | 8.63 | 1139 | 1123 |  |
| 19 | Propyl-2-Methyl butanoate | ester | 9.11 | 1154 | 1134 |  |
| 20 | Butyl propanoate | ester | 9.23 | 1157 | 1139 |  |
| 21 | 1-Butanol | alcohol | 9.36 | 1161 | 1142 |  |
| 22 | 2-Methylpropyl butanoate | ester | 9.78 | 1173 | 1158 | STD |
| 23 | Amyl acetate | ester | 10.34 | 1189 | 1176 | STD |
| 24 | Methyl hexanoate | ester | 10.78 | 1202 | 1184 |  |
| 25 | γ-Terpinene | terpene | 11.04 | 1210 | 1246 |  |
| 26 | Limonene | terpene | 11.12 | 1212 | 1200 |  |
| 27 | 1,8-Cineole | terpene | 11.27 | 1216 | 1213 |  |
| 28 | 2-Methyl-1-butanol | alcohol | 11.46 | 1222 | 1208 |  |
| 29 | (E)-2-Hexenal | aldehyde | 11.81 | 1231 | 1213 | STD |
| 30 | Butyl butanoate | ester | 11.83 | 1232 | 1220 |  |
| 31 | Butyl-2-methyl butanoate | ester | 12.25 | 1244 | 1235 | STD |
| 32 | Ethyl hexanoate | ester | 12.37 | 1248 | 1233 |  |
| 33 | 1-Dodecene | hydrocarbon | 12.42 | 1249 | 1243 |  |
| 34 | 3-Methyl-2-buten-1-ol acetate | ester | 12.90 | 1263 | 1249 |  |
| 35 | Pentanol | alcohol | 12.96 | 1265 | 1250 |  |
| 36 | Isoamyl butanoate | ester | 13.40 | 1277 | 1259 | STD |
| 37 | Hexyl acetate | ester | 13.72 | 1286 | 1272 | STD |
| 38 | 2-Octanone | ketone | 14.09 | 1297 | 1287 |  |
| 39 | 1-octen-3-one | ketone | 14.67 | 1314 | 1300 | STD |
| 40 | (E)-3-Hexenyl acetate | ester | 14.94 | 1322 | 1306 |  |
| 41 | Amyl butanoate | ester | 15.16 | 1328 | 1305 | STD |
| 42 | (Z)-3-Hexenyl acetate | ester | 15.21 | 1330 | 1315 |  |
| 43 | Propyl Hexanoate | ester | 15.23 | 1330 | 1316 |  |
| 44 | 2-Heptanol | alcohol | 15.25 | 1331 | 1320 |  |
| 45 | 3-Methyl-2-buten-1-ol | alcohol | 15.36 | 1334 | 1320 |  |
| 46 | (E)-2-Heptenal | aldehyde | 15.38 | 1335 | 1323 |  |
| 47 | 5-Hexenyl Acetate | ester | 15.54 | 1339 | 1313 |  |
| 48 | (E)-2-Hexenyl acetate | ester | 15.81 | 1347 | 1352 | STD |
| 49 | 6-methyl-5-hepten-2-one | ketone | 15.89 | 1350 | 1338 |  |
| 50 | n-Hexyl Isobutanoate | ester | 15.93 | 1351 | 1339 |  |
| 51 | Hexanol | alcohol | 16.45 | 1366 | 1355 | STD |
| 52 | (Z)-3-Hexen-1-ol | alcohol | 17.48 | 1396 | 1382 |  |
| 53 | Nonanal | aldehyde | 17.73 | 1404 | 1391 |  |
| 54 | 3-Octanol | alcohol | 17.74 | 1404 | 1393 |  |
| 55 | 2,4-Hexadienal (1) | aldehyde | 17.94 | 1410 | 1400 |  |
| 56 | 2,4-Hexadienal (2) | aldehyde | 18.05 | 1414 | 1400 |  |
| 57 | (E)-2-Hexen-1-ol | alcohol | 18.18 | 1418 | 1405 | STD |
| 58 | 5-Hexen-1-ol | alcohol | 18.31 | 1422 | 1394 |  |
| 59 | Butyl hexanoate | ester | 18.34 | 1423 | 1408 |  |
| 60 | Hexyl butanoate | ester | 18.43 | 1425 | 1414 | STD |
| 61 | 1-Tetradecene | hydrocarbon | 19.15 | 1447 | 1443 |  |
| 62 | (E)-Linalool oxide A | terpene | 19.20 | 1449 | 1452 |  |
| 63 | 1-octen-3-ol | alcohol | 19.58 | 1461 | 1450 |  |
| 64 | 1-Heptanol | alcohol | 19.78 | 1467 | 1453 |  |
| 65 | 6-methyl-5-hepten-2-ol | alcohol | 20.00 | 1474 | 1465 |  |
| 66 | Furfural | aldehyde | 20.07 | 1476 | 1461 |  |
| 67 | (Z)-Linalool oxide B | terpene | 20.11 | 1477 | 1444 |  |
| 68 | 3,4,5-Trimethyl-4-heptanol | alcohol | 20.72 | 1496 | n.a. |  |
| 69 | 2-ethyl-1-hexanol | alcohol | 20.90 | 1502 | 1491 |  |
| 70 | Decanal | aldehyde | 21.09 | 1508 | 1498 |  |
| 71 | Pentyl hexanoate | ester | 21.46 | 1520 | 1501 | STD |
| 72 | 2-Nonanol | alcohol | 21.76 | 1529 | 1521 |  |
| 73 | Benzaldehyde | aldehyde | 21.87 | 1533 | 1520 | STD |
| 74 | Furfuryl Acetate | ester | 22.38 | 1550 | 1531 |  |
| 75 | Linalool | terpene | 22.60 | 1557 | 1547 |  |
| 76 | Octanol | alcohol | 22.97 | 1569 | 1557 | STD |
| 77 | Hexyl hexanoate | ester | 24.45 | 1618 | 1602 |  |
| 78 | Butyrolactone | lactone | 25.03 | 1637 | 1632 |  |
| 79 | 1-Hexadecene | hydrocarbon | 25.33 | 1648 | 1648 |  |
| 80 | Acetophenone | ketone | 25.68 | 1659 | 1647 |  |
| 81 | Pentanoic Acid | acid | 25.81 | 1664 | 1733 |  |
| 82 | Nonanol | alcohol | 26.01 | 1671 | 1660 | STD |
| 83 | Furfuryl Alcohol | alcohol | 26.03 | 1672 | 1660 |  |
| 84 | p-Allyl anisole | phenylpropene | 26.29 | 1680 | 1670 |  |
| 85 | 2-Methyl Butanoic Acid | acid | 26.88 | 1701 | 1662 | STD |
| 86 | α-Terpineol | terpene | 26.97 | 1704 | 1697 |  |
| 87 | 5-ethyldihydro-2(3H)-furanone | lactone | 27.10 | 1709 | 1694 | STD |
| 88 | Butyl 3-hydroxybutanoate | ester | 27.25 | 1714 | 1684 |  |
| 89 | 3-(methylthio)-1-propanol | sulfur | 27.63 | 1728 | 1719 | STD |
| 90 | (Z,E)-α-Farnesene | sesquiterpene | 27.83 | 1735 | 1725 |  |
| 91 | Benzyl acetate | ester | 27.96 | 1740 | 1720 |  |
| 92 | (E,E)-α-Farnesene | sesquiterpene | 28.45 | 1757 | 1746 | STD |
| 93 | Dimethyl benzenemethanol | alcohol | 28.77 | 1769 | 1773 |  |
| 94 | (Z,Z)-α-Farnesene | sesquiterpene | 29.48 | 1795 | 1697 |  |
| 95 | 2-(2-butoxyethoxy)-ethanol | alcohol | 29.66 | 1801 | 1796 |  |
| 96 | (Z)-3-Decen-1-ol | alcohol | 29.83 | 1808 | 1789 |  |
| 97 | 2,4-Dimethyl Benzaldehyde | aldehyde | 30.18 | 1821 | 1726 |  |
| 98 | 2-Phenethyl Acetate | ester | 30.32 | 1826 | 1813 |  |
| 99 | β-Damascenone | C13-norisoprenoid | 30.40 | 1829 | 1823 |  |
| 100 | Anethol | terpene | 30.68 | 1840 | 1817 | STD |
| 101 | 1-Octadecene | hydrocarbon | 30.95 | 1850 | 1836 |  |
| 102 | Geranyl acetone | C13-norisoprenoid | 31.33 | 1864 | 1859 |  |
| 103 | Hexanoic Acid | acid | 31.83 | 1883 | 1846 |  |
| 104 | 2-phenylethanol | alcohol | 32.90 | 1925 | 1906 |  |
| 105 | Benzothiazole | heterocyclic | 33.90 | 1964 | 1958 |  |
| 106 | 2-Ethyl Hexanoic Acid | acid | 34.27 | 1979 | 1960 |  |
| 107 | 1-Dodecanol | alcohol | 34.31 | 1981 | 1966 |  |
| 108 | Phenol | alcohol | 35.26 | 2019 | 2000 |  |
| 109 | Isopropyl Myristate | ester | 35.89 | 2045 | 2027 |  |
| 110 | 1-methoxyethyl benzoate | ester | 36.98 | 2089 | n.a. |  |
| 111 | 4-Methyl Phenol | alcohol | 37.11 | 2095 | 2080 |  |
| 112 | Octanoic Acid | acid | 37.22 | 2099 | 2060 |  |
| 113 | 3-Methyl Phenol | alcohol | 37.30 | 2102 | 2091 |  |
| 114 | 2-phenoxyethanol | alcohol | 38.56 | 2135 | 2121 | STD |
| 115 | Nonanoic Acid | acid | 39.74 | 2166 | 2171 | STD |
| 116 | Methyl Palmitate | ester | 40.19 | 2178 | 2208 |  |
| 117 | Decanoic Acid | acid | 42.16 | 2238 | 2276 | STD |

RT: retention time (min); LRI_cal_: calculated linear retention index; LRI_nist_: Linear retention index reported in the MS-NIST database for a polar column; STD: compounds identified by authentic standard; n.a.: not available.

**Table S3. Characteristics of apple fruit and juice and their correlations with sensory attributes.**

|  | Mean | SD | Min | Max | Sweetness | | | | Sourness | | | | Astringency | | | |
| --- | --- | --- | --- | --- | --- | --- | --- | --- | --- | --- | --- | --- | --- | --- | --- | --- |
|  |  |  |  |  | r/𝝆 | *p* | Lower 95% CI | Upper 95% CI | r/𝝆 | *p* | Lower 95% CI | Upper 95% CI | r/𝝆 | p | Lower 95% CI | Upper 95% CI |
| *Apple fruit* |  |  |  |  |  |  |  |  |  |  |  |  |  |  |  |  |
| I_AD_ | 1.04 | 0.48 | 0.04 | 1.84 | 0.070 | 0.715 | -0.419 | 0.298 | 0.155 | 0.413 | -0.217 | 0.488 | 0.135 | 0.477 | -0.237 | 0.472 |
| Starch (Iodine test) | 8.5 | 1.8 | 3.0 | 10.0 | *-0.036* | 0.850 | -0.391 | 0.328 | *-0.111* | 0.560 | -0.453 | 0.260 | *-0.086* | 0.652 | -0.433 | 0.283 |
| DRR(°Brix) | 12.2 | 1.4 | 10.1 | 16.2 | *0.349* | 0.059 | -0.013 | 0.630 | *0.023* | 0.906 | -0.340 | 0.380 | *-0.091* | 0.633 | -0.437 | 0.279 |
| *Apple juice* |  |  |  |  |  |  |  |  |  |  |  |  |  |  |  |  |
| Glucose (g/L) | 24.1 | 5.1 | 17.0 | 37.0 | *0.102* | 0.590 | -0.268 | 0.446 | *-0.142* | 0.453 | -0.478 | 0.230 | *0.026* | 0.891 | -0.337 | 0.383 |
| Fructose (g/L) | 62.1 | 10.6 | 44.0 | 92.0 | 0.213 | 0.258 | -0.159 | 0.532 | 0.122 | 0.522 | -0.250 | 0.462 | 0.019 | 0.920 | -0.343 | 0.377 |
| Sucrose (g/L) | 34.2 | 14.2 | 3.4 | 68.0 | **0.479** | **0.007** | 0.144 | 0.716 | -0.034 | 0.859 | -0.389 | 0.330 | -0.271 | 0.148 | -0.575 | 0.099 |
| Xylose (g/L) | 0.55 | 0.27 | 0.28 | 1.34 | *-0.054* | 0.775 | -0.407 | 0.312 | *-0.167* | 0.377 | -0.497 | 0.205 | *0.010* | 0.959 | -0.352 | 0.369 |
| Sorbitol (g/L) | 5.4 | 2.7 | 1.7 | 13.6 | *-0.175* | 0.354 | -0.504 | 0.197 | *0.339* | 0.067 | -0.024 | 0.623 | ***0.364*** | **0.048** | 0.004 | 0.640 |
| Malic acid (g/L) | 8.02 | 3.70 | 3.25 | 19.40 | ***-0.634*** | **< .001** | -0.809 | -0.354 | ***0.880*** | **< .001** | 0.762 | 0.942 | ***0.792*** | **< .001** | 0.604 | 0.896 |
| Ascorbic acid (g/L) | 1.22 | 0.54 | 0.34 | 2.98 | *0.303* | 0.104 | -0.064 | 0.598 | *-0.030* | 0.875 | -0.386 | 0.334 | *-0.057* | 0.766 | -0.409 | 0.310 |
| Sweetness (0-100 scale) | 34.5 | 17.8 | 4.2 | 66.8 | _ | | | | _ | | | | _ | | | |
| Sourness (0-100 scale) | 52.1 | 25.2 | 11.1 | 92.5 |  |  |  |  |  |  |  |  |  |  |  |  |
| Astringency (0-100 scale) | 44.7 | 17.4 | 11.5 | 77.7 |  |  |  |  |  |  |  |  |  |  |  |  |

IAD: Index of Absorbance Difference; DRR (°Brix) dry refractometric residue; SD: Standard deviation. Significant correlations (a of 0.05) are marked in bold. Monotonic (ρ) relationships are in Italic.

**Table S4. Polyphenols and correlation with sensory attributes.**

|  | Mean | SD | Min | Max | Sweetness | | | | Sourness | | | | Astringency | | | |
| --- | --- | --- | --- | --- | --- | --- | --- | --- | --- | --- | --- | --- | --- | --- | --- | --- |
|  |  |  |  |  | r/𝝆 | *p* | Lower 95% CI | Upper 95% CI | r/𝝆 | *p* | Lower 95% CI | Upper 95% CI | r/𝝆 | p | Lower 95% CI | Upper 95% CI |
| *Polyphenols* (𝝻g/g) |  |  |  |  |  |  |  |  |  |  |  |  |  |  |  |  |
| 4-Aminobenzoic acid | 0.039 | 0.023 | 0.010 | 0.098 | *0.083* | 0.663 | -0.286 | 0.430 | -0.08 | 0.695 | -0.424 | 0.293 | -0.32 | 0.089 | -0.607 | 0.050 |
| Vanillin | 0.044 | 0.027 | 0.011 | 0.119 | *-0.1* | 0.595 | -0.445 | 0.269 | *-0.06* | 0.770 | -0.408 | 0.311 | *-0.08* | 0.669 | -0.429 | 0.287 |
| Vanillic acid | 0.038 | 0.149 | 0.000 | 0.814 | *-0.3* | 0.108 | -0.595 | 0.068 | *0.237* | 0.208 | -0.135 | 0.550 | *0.285* | 0.127 | -0.084 | 0.585 |
| Caffeic acid | 0.16 | 0.39 | 0.00 | 1.64 | *-0.2* | 0.300 | -0.519 | 0.177 | *-0.009* | 0.964 | -0.368 | 0.353 | *0.014* | 0.942 | -0.348 | 0.372 |
| Neochlorogenic acid | 0.34 | 0.57 | 0.04 | 3.23 | *-0.29* | 0.122 | -0.587 | 0.080 | *0.187* | 0.323 | -0.186 | 0.513 | *0.281* | 0.132 | -0.088 | 0.582 |
| Cryptochlorogenic acid | 8.9 | 11.8 | 0.81 | 65.8 | *-0.3* | 0.105 | -0.597 | 0.065 | *0.231* | 0.219 | -0.141 | 0.546 | *0.309* | 0.097 | -0.058 | 0.602 |
| Chlorogenic acid | 122 | 88.3 | 10.8 | 299 | ***-0.42*** | **0.022** | -0.676 | -0.068 | 0.343 | 0.064 | -0.020 | 0.626 | **0.383** | **0.037** | 0.027 | 0.653 |
| Catechin | 5.4 | 6.4 | 0.0 | 29.3 | *-0.11* | 0.549 | -0.456 | 0.257 | *0.054* | 0.779 | -0.313 | 0.406 | *0.058* | 0.760 | -0.308 | 0.410 |
| Epicatechin | 31.5 | 32.5 | 0.5 | 170 | *-0.35* | 0.059 | -0.629 | 0.014 | *0.224* | 0.235 | -0.149 | 0.540 | *0.316* | 0.089 | -0.050 | 0.607 |
| Procyanidin B1 | 27.4 | 27.5 | 0.0 | 107 | *-0.27* | 0.145 | -0.576 | 0.097 | *0.206* | 0.276 | -0.167 | 0.527 | *0.238* | 0.205 | -0.134 | 0.551 |
| Procyanidin B2 + B4 (as B2) | 119 | 146 | 0.12 | 685 | ***-0.38*** | **0.041** | -0.648 | -0.017 | *0.267* | 0.154 | -0.103 | 0.572 | ***0.367*** | **0.046** | 0.008 | 0.642 |
| Quercetin-3-Rha | 0.62 | 1.09 | 0.01 | 5.73 | *-0.19* | 0.305 | -0.518 | 0.179 | *0.259* | 0.166 | -0.111 | 0.567 | *0.276* | 0.141 | -0.094 | 0.578 |
| Quercetin-3-Glc + Quercetin-3-Gal (As Que-3-Glc) | 2.1 | 3.2 | 0.0 | 17.1 | ***-0.4*** | **0.019** | -0.690 | -0.080 | ***0.378*** | **0.026** | 0.056 | 0.678 | ***0.462*** | **0.006** | 0.166 | 0.733 |
| *Dihydrochalcones* (𝝻g/g) |  |  |  |  |  |  |  |  |  |  |  |  |  |  |  |  |
| Phlorizin | 1.9 | 5.6 | 0.0 | 30.3 | *-0.330* | 0.075 | -0.617 | 0.035 | *0.297* | 0.111 | -0.071 | 0.594 | ***0.372*** | **0.043** | 0.013 | 0.646 |
| Phloretin Xylo Glc | 6.3 | 10.5 | 0.4 | 56.1 | ***-0.46*** | **0.010** | -0.706 | -0.125 | ***0.432*** | **0.017** | 0.086 | 0.686 | ***0.502*** | **0.004** | 0.182 | 0.734 |

Rha: rhamninoside; Glc: glucoside; Ga: galactoside; Que: quercetin; Xylo Glc: xylo-glucoside. Significant correlations (α of 0.05) are marked in bold. Monotonic (ρ) relationships are in Italic.

**Table S5. Volatiles organic compounds significantly correlated (a of 0.05) with sensory attributes.**

| Volatile compounds (μg/L) | Mean | SD | Min | Max | Sweetness | | | | Sourness | | | | Astringency | | | |
| --- | --- | --- | --- | --- | --- | --- | --- | --- | --- | --- | --- | --- | --- | --- | --- | --- |
|  |  |  |  |  | r/𝝆 | *p* | Lower 95% CI | Upper 95% CI | r/𝝆 | *p* | Lower 95% CI | Upper 95% CI | r/𝝆 | p | Lower 95% CI | Upper 95% CI |
| ***Esters*** |  |  |  |  |  |  |  |  |  |  |  |  |  |  |  |  |
| Propyl acetate | 217 | 403 | n.d. | 1502 | *0.629* | 0.001 | 0.348 | 0.807 | - | - | - | - | *-0.403* | 0.027 | -0.667 | -0.050 |
| Isobutyl acetate | 241 | 296 | n.d. | 1094 | *0.522* | 0.003 | 0.200 | 0.743 | - | - | - | - | - | - | - | - |
| Propyl propanoate | 119 | 385 | n.d. | 2041 | *0.442* | 0.014 | 0.098 | 0.692 | - | - | - | - | - | - | - | - |
| Butyl acetate | 9629 | 11228 | 72.7 | 35572 | 0.511 | 0.004 | 0.185 | 0.736 | - | - | - | - | -0.518 | 0.003 | -0.740 | -0.195 |
| Isoamyl acetate | 12496 | 15499 | 27.6 | 50459 | *0.475* | 0.008 | 0.138 | 0.713 | - | - | - | - | - | - | - | - |
| Propyl butanoate | 1288 | 3505 | n.d. | 17190 | *0.427* | 0.018 | 0.079 | 0.683 | - | - | - | - | - | - | - | - |
| Propyl-2-methyl butanoate | 235 | 570 | n.d. | 2544 | *0.452* | 0.012 | 0.109 | 0.698 | - | - | - | - | - | - | - | - |
| Amyl acetate | 2065 | 2858 | 8.88 | 11596 | *0.577* | 0.001 | 0.274 | 0.776 | - | - | - | - | - | - | - | - |
| Butyl butanoate | 4941 | 5267 | 114 | 18151 | 0.412 | 0.024 | 0.061 | 0.673 | - | - | - | - | *-0.528* | 0.003 | -0.746 | -0.207 |
| Butyl-2-methyl butanoate | 589 | 903 | 3.53 | 3533 | *0.539* | 0.002 | 0.222 | 0.753 | - | - | - | - | *-0.428* | 0.018 | -0.683 | -0.080 |
| Isoamyl butanoate | 45.2 | 62.9 | n.d. | 245 | *0.383* | 0.036 | 0.027 | 0.653 | - | - | - | - | *-0.367* | 0.046 | -0.642 | -0.007 |
| Hexyl acetate | 22900 | 33730 | 138 | 1E+05 | 0.534 | 0.002 | 0.216 | 0.750 | - | - | - | - | *-0.427* | 0.018 | -0.683 | -0.079 |
| Propyl hexanoate | 154 | 412 | n.d. | 1977 | *0.442* | 0.014 | 0.097 | 0.692 | - | - | - | - | - | - | - | - |
| (Z)-3-Hexen-1-ol acetate | 399 | 848 | n.d. | 3972 | *0.404* | 0.027 | 0.052 | 0.667 | - | - | - | - | - | - | - | - |
| (E)-2-hexenyl acetate | 79.9 | 104 | 1.13 | 397 | *0.477* | 0.008 | 0.141 | 0.714 | *-0.380* | 0.038 | -0.651 | -0.023 | *-0.39* | 0.032 | -0.659 | -0.037 |
| Butyl hexanoate | 134 | 166 | n.d. | 616 | *0.388* | 0.034 | 0.033 | 0.657 | *-0.385* | 0.036 | -0.655 | -0.029 | *-0.5* | 0.005 | -0.726 | -0.165 |
| Hexyl butanoate | 1989 | 3074 | 9.95 | 13008 | *0.444* | 0.014 | 0.099 | 0.693 | - | - | - | - | *-0.43* | 0.019 | -0.682 | -0.079 |
| Hexyl hexanoate | 168 | 227 | 11.1 | 1035 | *0.381* | 0.038 | 0.024 | 0.652 | - | - | - | - | *-0.460* | 0.011 | -0.704 | -0.119 |
| Butyl-3-hydroxybutanoate | 22 | 19.5 | n.d. | 77.1 | 0.615 | 0.001 | 0.327 | 0.798 | -0.547 | 0.002 | -0.758 | -0.233 | -0.657 | 0.001 | -0.822 | -0.388 |
| Hexyl (E)-2-methyl-2-butenoate | 4.18 | 7.58 | n.d. | 38.7 | - | - | - | - | *-0.484* | 0.007 | -0.719 | -0.150 | *-0.55* | 0.002 | -0.761 | -0.24 |
| 2-Phenethyl acetate | 11.4 | 29.5 | n.d. | 162 | *0.377* | 0.040 | 0.019 | 0.649 | - | - | - | - | - | - | - | - |
| (E)-3-Hexenyl acetate | 18.1 | 25.2 | n.d. | 84.6 | - | - | - | - | - | - | - | - | *-0.43* | 0.018 | -0.683 | -0.081 |
| Isopropyl myristate | 10.7 | 3.69 | 4.98 | 19.6 | 0.402 | 0.028 | 0.049 | 0.666 | -0.393 | 0.032 | -0.660 | -0.038 | -0.442 | 0.015 | -0.692 | -0.097 |
| 1-Methoxyethyl benzoate | 9.68 | 1.99 | 6.5 | 15.1 | *0.511* | 0.004 | 0.184 | 0.736 | - | - | - | - | *-0.48* | 0.008 | -0.714 | -0.139 |
| ***Aldehydes*** |  |  |  |  |  |  |  |  |  |  |  |  |  |  |  |  |
| Butanal | 31.3 | 44.5 | n.d. | 200 | *0.391* | 0.033 | 0.036 | 0.659 | *-0.375* | 0.041 | -0.648 | -0.017 | *-0.46* | 0.011 | -0.702 | -0.116 |
| Hexanal | 5023 | 3584 | 192 | 13013 | 0.704 | 0.001 | 0.460 | 0.849 | -0.534 | 0.002 | -0.750 | -0.216 | *-0.6* | 0.001 | -0.79 | -0.308 |
| 2-Methyl-2-butenal | 14.2 | 35.3 | n.d. | 194 | *-0.470* | 0.009 | -0.710 | -0.132 | *0.412* | 0.024 | 0.060 | 0.672 | *0.469* | 0.009 | 0.131 | 0.709 |
| (E)-2-Hexenal | 5469 | 4961 | 392 | 19516 | *0.612* | 0.001 | 0.323 | 0.797 | *-0.513* | 0.004 | -0.737 | -0.188 | *-0.45* | 0.013 | -0.696 | -0.104 |
| 2,4-Hexadienal (E,E) | 28 | 28.6 | 3.9 | 141 | *0.482* | 0.007 | 0.148 | 0.718 | *-0.603* | 0.001 | -0.791 | -0.310 | *-0.42* | 0.020 | -0.679 | -0.072 |
| 2,4-Hexadienal (E,Z) | 64.7 | 64.3 | 8.49 | 287 | *0.508* | 0.004 | 0.181 | 0.734 | *-0.610* | 0.001 | -0.795 | -0.319 | *-0.44* | 0.015 | -0.691 | -0.095 |
| 2,4-Dimethyl benzaldehyde | 17.9 | 4.98 | 9.16 | 29.4 | 0.547 | 0.002 | 0.233 | 0.758 | - | - | - | - | -0.361 | 0.050 | -0.638 | -0.001 |
| ***Alcohols*** |  |  |  |  |  |  |  |  |  |  |  |  |  |  |  |  |
| 2-Heptanol | 48.2 | 105 | n.d. | 415 | *0.485* | 0.007 | 0.151 | 0.720 | - | - | - | - | - | - | - | - |
| Hexanol | 22179 | 14435 | 3395 | 54882 | 0.390 | 0.033 | 0.035 | 0.658 | -0.368 | 0.045 | -0.643 | -0.009 | -0.558 | 0.001 | -0.765 | -0.248 |
| Nonanol | 30.8 | 12.3 | 13.2 | 62.7 | - | - | - | - | -0.437 | 0.016 | -0.689 | -0.091 | -0.486 | 0.006 | -0.72 | -0.152 |
| 2-Nonanol | 47 | 106 | n.d. | 463 | *0.513* | 0.004 | 0.188 | 0.737 | - | - | - | - | - | - | - | - |
| 1-Butanol | 2554 | 2858 | 129 | 12263 | - | - | - | - | *-0.533* | 0.002 | -0.749 | -0.213 | *-0.61* | 0.001 | -0.795 | -0.318 |
| Pentanol | 261 | 175 | 47.4 | 748 | - | - | - | - | *-0.419* | 0.021 | -0.677 | -0.070 | *-0.51* | 0.004 | -0.733 | -0.179 |
| 1-Dodecanol | 28.3 | 8.22 | 14.7 | 40.9 | - | - | - | - | - | - | - | - | -0.363 | 0.049 | -0.64 | -0.003 |
| ***Ketones*** |  |  |  |  |  |  |  |  |  |  |  |  |  |  |  |  |
| 2-Octanone | 515 | 55.3 | 377 | 630 | -0.383 | 0.037 | -0.653 | -0.026 | 0.441 | 0.015 | 0.096 | 0.692 | - | - | - | - |
| ***Acids*** |  |  |  |  |  |  |  |  |  |  |  |  |  |  |  |  |
| 2-Methylbutanoic acid | 31.6 | 79.2 | n.d. | 405 | *0.439* | 0.015 | 0.094 | 0.690 | - | - | - | - | *-0.4* | 0.031 | -0.661 | -0.04 |
| Pentanoic acid | 8.39 | 10.8 | n.d. | 33.6 | - | - | - | - | - | - | - | - | *-0.51* | 0.004 | -0.742 | -0.183 |
| Hexanoic acid | 62.6 | 32 | 26.1 | 143 | *0.499* | 0.005 | 0.169 | 0.728 | *-0.598* | 0.001 | -0.788 | -0.302 | *-0.64* | 0.001 | -0.814 | -0.366 |
| Octanoic acid | 36.4 | 22.4 | 11.6 | 133 | *0.468* | 0.009 | 0.129 | 0.708 | *-0.368* | 0.045 | -0.643 | -0.009 | *-0.47* | 0.009 | -0.709 | -0.130 |
| Dodecanoic acid | 27.4 | 8.21 | 16.3 | 50.2 | 0.417 | 0.022 | 0.066 | 0.676 | - | - | - | - | - | - | - | - |
| ***Terpenes and derivatives*** |  |  |  |  |  |  |  |  |  |  |  |  |  |  |  |  |
| α-Terpineol | 10.7 | 6.8 | 4.75 | 35.8 | *0.522* | 0.003 | 0.200 | 0.743 | - | - | - | - | - | - | - | - |
| (Z,E) -α-Farnesene | 16.1 | 14.1 | 2.47 | 63.9 | *0.383* | 0.037 | 0.026 | 0.653 | - | - | - | - | - | - | - | - |
| p-Allylanisole | 286 | 553 | 12.1 | 2028 | *0.402* | 0.028 | 0.048 | 0.666 | - | - | - | - | - | - | - | - |
| Anethole | 31.2 | 26.6 | 7.82 | 110 | *0.391* | 0.033 | 0.035 | 0.658 | - | - | - | - | - | - | - | - |
| β-Damascenone | 64.7 | 46.8 | 4.43 | 233 | - | - | - | - | *0.525* | 0.003 | 0.204 | 0.745 | *0.395* | 0.031 | 0.04 | 0.661 |
| ***Others*** |  |  |  |  |  |  |  |  |  |  |  |  |  |  |  |  |
| 1-Hexadecene | 225 | 57.4 | 138 | 370 | - | - | - | - | - | - | - | - | -0.388 | 0.034 | -0.657 | -0.033 |
| 5-Ethyldihydro-2(3H)-furanone | 6.85 | 6.42 | n.d. | 25.5 | *0.418* | 0.021 | 0.068 | 0.677 | - | - | - | - | *-0.52* | 0.003 | -0.741 | -0.196 |
| Benzothiazole | 4.48 | 2.34 | 1.71 | 11.0 | *0.552* | 0.002 | 0.240 | 0.761 | - | - | - | - | *-0.46* | 0.010 | -0.706 | -0.125 |

Monotonic (ρ) relationships are in Italic.
