# Supplementary material for: Chemical Drivers of Sweetness, Sourness, and Astringency in Apple Juice Identified Using Support Vector Machine Regression
Source: J Food Sci. 2026 Sep 16;91(9):e71479. doi: 10.1111/1750-3841.71479 (PMC13579707; doi:10.1111/1750-3841.71479)
Supplement: Supplementary file 2 [file JFDS-91-0-s002.docx]

**Figure S1**. Performance of the sensory panel displayed as p-value vs. MSE plots for the attributes Astringent, Sour and Sweet. Each point represents one of the 14 trained assessors (G1–G14). The x-axis reports the mean squared error (MSE) of the assessor, used as an index of repeatability, while the y-axis reports the p-value of the product effect from a two-way ANOVA, used as an index of discrimination ability. The red dashed line marks the p = 0.05 significance threshold, and the shaded band identifies the region of significant discrimination. Point shape denotes discrimination ability: circles indicate assessors with p < 0.05 (significant discrimination), whereas triangles indicate assessors with p ≥ 0.05. Point colour denotes repeatability relative to the panel: blue indicates assessors with an MSE below the panel mean, whereas red indicates an MSE above the panel mean. Assessors located in the lower-left region (green area) combine good repeatability with significant discrimination and represent the optimal performance profile.

**Figure S2**. Tucker-1 correlation loading plots describing agreement for the attributes Astringent (A), Sour (B) and Sweet (C). Plots are based on a principal component analysis of the assessor scores, with the first two components (PC1 = 91.6%, PC2 = 8.4%) shown and the two concentric circles correspond to 50% (inner) and 100% (outer) of explained variance. Each point represents one of the 14 trained assessors (G1–G14), and the green point denotes the panel consensus (the panel-average configuration). Point colour encodes the distance of each assessor from the consensus: blue indicates a low distance, purple an intermediate distance, and red a high distance.

**Figure S3**. Examples of Predicted versus Observed values from SVMR for sweetness (A, R^2^CV = 0.615, RMSECV = 10.6), sourness (B, R^2^CV = 0.746, RMSECV = 11.2) and astringency (C, R^2^CV = 0.620, RMSECV = 9.4).

**Figure S4**. Beeswarm plot for Shapley Values for sweetness (see SVMR model in **Figure S3**).

**Figure S5**. Beeswarm plot for Shapley Values for sourness (see SVMR model in **Figure S3**).

**Figure S6**. Beeswarm plot for Shapley Values for astringency (see SVMR model in **Figure S3**).
